# ScatterHough: Automatic Lane Detection from Noisy LiDAR Data

**DOI:** 10.3390/s22145424

**Published:** 2022-07-20

**Authors:** Honghao Zeng, Shihong Jiang, Tianxiang Cui, Zheng Lu, Jiawei Li, Boon-Giin Lee, Junsong Zhu, Xiaoying Yang

**Affiliations:** 1 School of Computer Science, University of Nottingham Ningbo China, Ningbo 315100, China; scyhz6@nottingham.edu.cn (H.Z.); zheng.lu@nottingham.edu.cn (Z.L.); jiawei.li@nottingham.edu.cn (J.L.); boon-giin.lee@nottingham.edu.cn (B.-G.L.); junsong.zhu@nottingham.edu.cn (J.Z.); scyxy3@nottingham.edu.cn (X.Y.); 2 Huawei Technologies Co., Ltd., Shanghai 201206, China; jiangshihong1020@163.com

**Keywords:** Hough Transform, curve fitting, scatter data, LiDAR point cloud

## Abstract

Lane detection plays an essential role in autonomous driving. Using LiDAR data instead of RGB images makes lane detection a simple straight line, and curve fitting problem works for realtime applications even under poor weather or lighting conditions. Handling scatter distributed noisy data is a crucial step to reduce lane detection error from LiDAR data. Classic Hough Transform (HT) only allows points in a straight line to vote on the corresponding parameters, which is not suitable for data in scatter form. In this paper, a Scatter Hough algorithm is proposed for better lane detection on scatter data. Two additional operations, ρ neighbor voting and ρ neighbor vote-reduction, are introduced to HT to make points in the same curve vote and consider their neighbors’ voting result as well. The evaluation of the proposed method shows that this method can adaptively fit both straight lines and curves with high accuracy, compared with benchmark and state-of-the-art methods.

## 1. Introduction

Over the last decade, autonomous driving has attracted more and more attention in both the academic community and automobile industry. Advanced Driver Assistance Systems (ADAS) become more and more intelligent providing assistance in daily driving or even taking an active part in less complicated situations such as highways or close loop scenarios. Among all the subsystems or algorithms in ADAS, automatic lane detection is essential for keeping vehicles safe and making road users better.

While lanes are usually easily identified by human drivers, automatic detection of lanes under all conditions are not as simple as many may think. Lane marking may be faded in terms of color or texture, after years of use or lack of maintenance. Some part of lane marks may be blocked or washed away due to road works. To make the situation worse, under poor weather conditions such as heavy rain or snow or low light conditions such as mid night, lane marks may be even hard for naked human eyes. Traditional RGB based lane detection methods [1,2,3,4,5,6] often fail to provide accurate results in these situations, even with the help of popular deep-learning based techniques [7,8].

To tackle such challenge, in addition to normal RGB cameras, many modern vehicles are equipped with LiDAR sensors for more robust data input in real time. It is shown that, based on LiDAR data, simple line or curve fitting that is robust to noises could produce practical lane detection performance in real time [9,10,11]. Such line fitting methods assume lanes are straight or curves and use different function prototypes for the fitting such as polynomial or Bezier curves of various orders [7,12].

Two commonly used methods in the fitting of noise data in the area of Computer Vision are RANdom SAmple Consensus (RANSAC) [13] and Hough Transform (HT) [14,15]. In terms of straight line fitting, RANSAC randomly samples data to estimate model parameters. The data points are then divided into two sets, inliers and outliers, according to whether they locate in the neighborhood of the model. A good estimation of parameters makes most sample data allocated in the inlier set. RANSAC does not guarantee the optimal solution because of limited sampling. Curve fitting has a high degree of freedom, and it is more susceptible to noise. In practice, it is difficult for RANSAC to obtain an optimal solution in curve fitting. In contrast, HT searches for the optimal solution by voting in the quantified model parameter space. Only the points located in a straight line could vote for the parameters. Other points do not contribute to voting, even for those in the neighborhood of the straight line. In curve fitting, the computational complexity of HT increases exponentially with the number of parameters, greatly reducing the computational efficiency.

Valid data points fluctuate around the true value due to noise. We call them scatter points. Data points in the neighborhood of estimated value should participate in the evaluation of the model. In order to obtain an optimal solution of the scatter fitting, we proposed a Scatter Hough algorithm for automatic lane detection, which allows the points in the neighborhood of estimated value to vote. In terms of curve fitting, the adaptive line segment is used to fit the curve, the candidate points are extracted around the straight line, and the curve parameters are obtained by fitting the candidate points by the least square method. Because the parameter space has only two dimensions of rho-theta, computational complexity is also reduced. In this way, our method is able better detect lanes in a straight line or a curve given noisy LiDAR data, often the case in real world situations. Experimental results on a popular real world dataset, PandaSet, show that Scatter Hough has a better performance in line fitting compared with RANSAC and original HT. At the same time, the adaptive line segment fitting curve can also achieve good performance in curve fitting. The contributions of this work are summarized as follows:

- ρ neighbor voting method is introduced into HT to allow points in the neighborhood of estimated value to vote, tailed for scatter points;
- ρ neighbor vote-reduction method is introduced into HT to drop votes that already contribute to existing fitted lines for better curve fitting;
- Experimental results on the popular PandaSet demonstrate that our method achieves better performance compared with other line fitting approaches.

## 2. Related Work

Lane detection plays an important role in autonomous driving. Traditional lane detection techniques use RGB images as input and are often based on handcrafted features and algorithms [2,3,4,5]. The work from Kluge and Lakshmanan [1] is the first attempt to design a lane detection model from RGB images, using features such as color, shape, and texture. The authors use clustering techniques to remove noises for better detection. In addition to these features, Hur et al. [6] use optical flow calculated from RGB images to further improve detection performance. While producing reasonable results, these handcrafted feature based methods often suffer from poor road conditions including lane variations due to different country regulations, poor weather conditions such as rain or snow, blurry images due to poor lighting condition, or large movement due to vehicle speeding. These practical issues make traditional lane detection techniques almost impossible to use in real world situations.

In recent years, with the fast development of deep-learning based techniques, many works attempt to detect lanes using techniques such as Convolutional Neural Networks (CNN). One of the representative deep-learning based methods is the work from Huval et al. [7], which uses CNN as a backbone to extract learnable features from RGB input, a similar model as to RCNN [16,17]. The method firstly divides the input image into grids and then uses a sliding window to generate multiple proposal bounding boxes which are regressed and classified for lane generation. Such methods produce excellent results when the input images are clear. However, because RGB images are the only input of the model, the detection accuracy is greatly affected by poor weather and lighting condition, or when the target is distant [8]. Moreover, such model is resource demanding, including both time and computational power due to factors such as the sliding window approach, deep neural networks, and DBSCAN clustering. Such a strong demand in time and computational power makes the model almost impossible to use in modern ADAS. Some experts suggest using cloud-edge computing to release the computational burden. The idea is that the autonomous vehicles are only responsible for sampling data, while the server may undertake the heavy computations and send back the results via the Internet. This is a good development direction, but mobile networks sometimes can have very large network latencies, and they may potentially cause serious damage [18].

Due to poor robustness and a large amount of calculation from these models, many works have started to use LiDAR data as the main input. LiDAR was initially designed to be equipped on trucks since they usually have a higher height that allows LiDAR to better perceive the surrounding environment. Generally speaking, LiDAR that is equipped on a 4 m-high truck can reach a detection range of 150 m [19]. Linder et al. [20] propose to use input from a LiDAR sensor to determine which part of road is the lane based on the different sensing values returned from surfaces of different materials. Compared with RGB based methods, such methods are not affected by weather and lighting conditions at all and little affected by distance. Furthermore, due to the quality of LiDAR data, deep neural networks are not needed to provide better detection accuracy. It is shown that simple line fitting algorithms with strong noise removal can produce satisfactory results in real world situations [9,10,11].

Generally, LiDAR-based lane detection methods can be decomposed into two main steps. The first step is to use LiDAR to extract geometric or physical features, and the second step is to fit the lane line using the extracted discrete data. In the first step, the raw physical information such as echo width and reflection intensity will be summarized into histograms, and data clustering algorithms such as DBSCAN will be applied to minimize the cluster variance and accordingly calculate the segmentation threshold of pavement and lane lines. Such approaches normally are very fast, but they are not robust due to lots of interference values (abnormal points) produced in the process [18]. One way to reduce the effects of abnormal points is to use GPS positioning, but this may require additional systems and a certain delay can be caused because of the network bandwidth [21]. The data fusion framework can be also adopted. For example, Zheng et al. [4] uses a deep learning model to find the mapping relationship between the LiDAR point cloud and camera RGB data, and make their semantics complementary. Although it can offer a higher clustering accuracy, the expensive computational cost makes it almost impossible to be deployed in practice. In the second step, a traditional approach focuses on polynomial fitting [9,10,21,22]. These methods assume lanes are straight lines or curves and use different function prototypes for the fitting such as polynomial or Bezier curves of various orders [7,12]. Algorithms such as RANSAC, DBSCAN, and their variants [13,23,24,25,26,27,28,29] are usually used to reduce effects from noises captured from the data. Tabelini et al. [22] and Li et al. [9] attempt to use polynomial line fit with neural networks trying to balance good fitting performance and computational demand. In this work, we mainly focus on the second step of LiDAR-based lane detection and propose a modified Hough method to transfer data points from the image space to parameter space for better lane detection accounting for noisy data.

## 3. The Proposed ScatterHough

Hough Transformation initially used the two-dimensional slope-intercept plane as the parameter space to detect lines [14]. Lines were detected by voting in the quantified parameter space, but the chosen plane was unbounded, which made it hard to be used in practice. Duda et al. [15] replaced the original slope–intercept plane with the ρ-θ plane with a bounded θ range, and further simplified the computation. This modification is now considered as the classic implementation of HT, which is still being widely adopted in practice. In this classic HT, lines are specified by the polar(norm) representation $\rho =x_{i}\;cos\theta +y_{i}\;sin\theta$, where ρ is the distance from the origin to the closest point on the straight line, and θ is the angle between the *x*-axis and the line connecting the origin with that closest point. For any point $\left(x_{i},y_{i}\right)$ on the line, ρ and θ remain constant, and each point in the xy-plane should give a sinusoid in the polar Hough parameter ρθ-space. Each curve in the Hough space represents the family of lines that pass through a particular point $\left(x_{i},y_{i}\right)$ in the xy-plane.

Hough transform is performed by quantizing the Hough space into finite intervals, known as accumulator cells. As it runs, each $\left(x_{i},y_{i}\right)$ is transformed into a discretized (ρ,θ) curve, and the accumulator cells which lie along this curve are incremented. By looking for local maxima in the accumulator space, the most likely lines can be extracted.

In real world scenarios, data points are often accompanied by noise and are distributed in the form of scattered points in space. The Hough transform makes the points on the same line/curve vote, and the points in the neighborhood do not participate in the voting, which is not suitable for the situation where the data are presented as scattered points. To resolve this issue, we propose two additional steps, ρ neighbor voting and ρ neighbor vote-reduction, in order to reduce the effect of the scatter-distributed noises.

### 3.1. ρ Neighbor Voting

**Figure 1:**
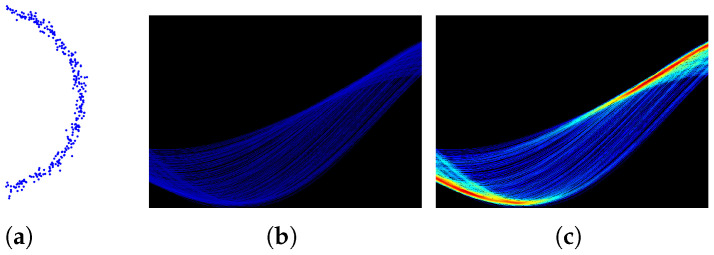
ρ Neighbor Voting. For (**b**,**c**), the *x*-axis is θ and the *y*-axis is ρ, and the colors with the deeper depth represent the bigger numbers of votes. (**a**) original data; (**b**) classic HT voting in Hough space; (**c**) ScatterHough voting in Hough space.

After simple accumulation in the classic HT, a cluster operation is performed on each non-empty ρ-θ cell $A_{\rho \theta }$ in the accumulator array *A* to add up all its neighbors’ votes in the neighbor offset range [−d,d]. The number of clustered votes will then be recorded. This operation is described in Equation (1), as shown in Figure 1c. The implementation details can be found in Algorithm 1:

(1) $$A_{\rho \theta }^{\prime }=\sum_{\rho_{i}=\rho -d}^{\rho +d}A_{\rho_{i}\theta }$$

**Figure 2:**
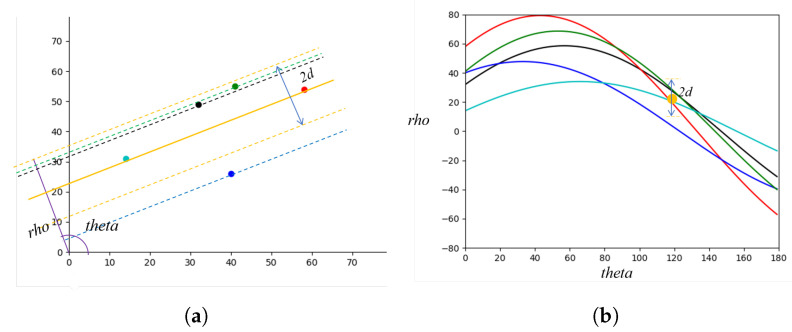
ρ Neighborhood. (**a**) neighborhood under Cartesian coordinates, each dot (in different colors) is a candidate Cartesian point and two candidate Cartesian points determine a fitted line; (**b**) neighborhood under Hough space, each dot corresponds to one fitted line (in same color) in Cartesian space and each curve represents all the possible lines that passing through one candidate Cartesian point (in same color).

As illustrated in Figure 2, the neighbors are the points $\left(x_{i},y_{i}\right)$ with distances to the line (ρ,θ) less than a given distance *d* in Cartesian coordinates. In the Hough space, these neighbors are equivalent to the curves that follow:

(2) $$\left|\;x_{i}\sin\theta +y_{i}\cos\theta -\rho \;\right|<d$$

**Algorithm 1:**ρ Neighbor Voting.
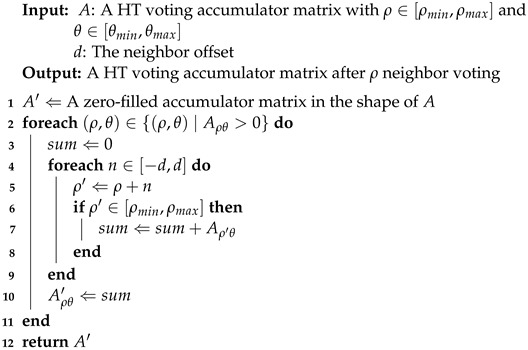


### 3.2. ρ Neighbor Vote-Reduction

In the clustered Hough space, only the ρ-θ pair with the highest vote number will be picked. All of its neighbors that contribute to this vote will then be dropped from the search space. The contributions to other votes by these dropped neighbors will also be removed. Such an operation ensures that each point belongs to one straight line only. The implementation details can be found in Algorithm 2.

**Figure 3:**
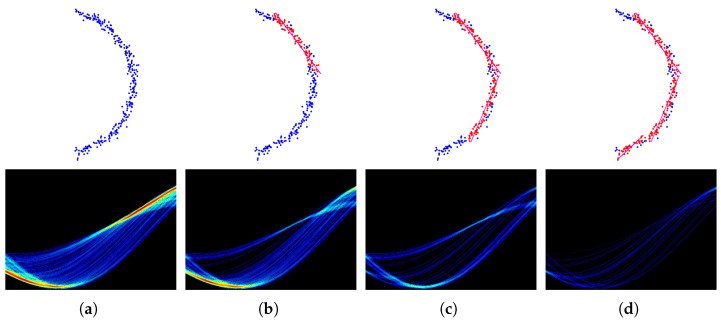
ρ Neighbor Vote-reduction. The top line, from left to right: original data, first line fit, second line fit, and third line fit. The bottom line, from left to right, first iteration of the proposed method in Hough space, second iteration of the proposed method in Hough space, third iteration of the proposed method in Hough space, fourth iteration of the proposed method in Hough space, the *x*-axis is θ and the *y*-axis is ρ, and the colors with the deeper depth represent the bigger numbers of votes. (**a**) 1st iteration; (**b**) 2nd iteration; (**c**) 3rd iteration; (**d**) 4th iteration.

Figure 3 shows the iterative process of the straight line fitting by using ρ neighbor voting and ρ neighbor vote-reduction.

Since the highest vote is used for each step to find the fitted lines instead of simply using a fixed threshold, such an operation can be adapted to fit curve scatters as well.
**Algorithm 2:**ρ Neighbor Vote-reduction.
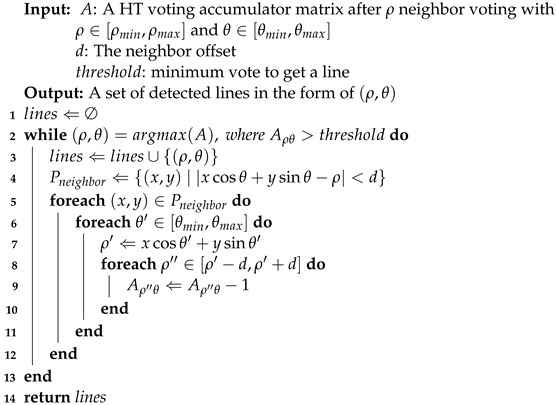


## 4. Evaluation

### 4.1. Dataset

The dataset we used is PandaSet maintained by Scale AI (https://scale.com/resources/download/pandaset, accessed on 24 March 2022.). It contains a variety of scene data, including various types of streets, intersections, circular islands, and viaducts with high-quality supporting annotations.

### 4.2. Experimental Results

**Figure 4:**
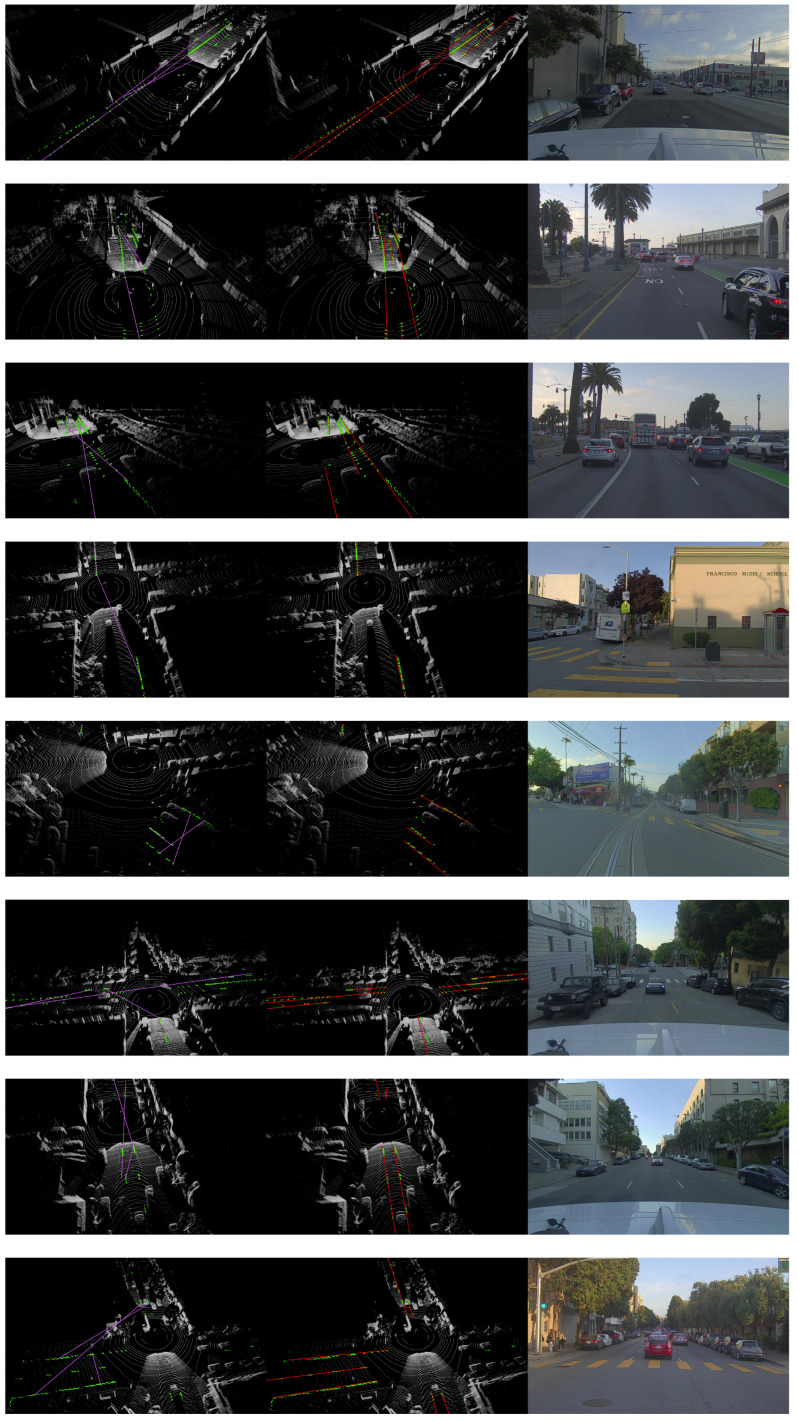
Visualization results for all scenes, from left to right: multiRansac, Ours, RGB.

**Figure 5:**
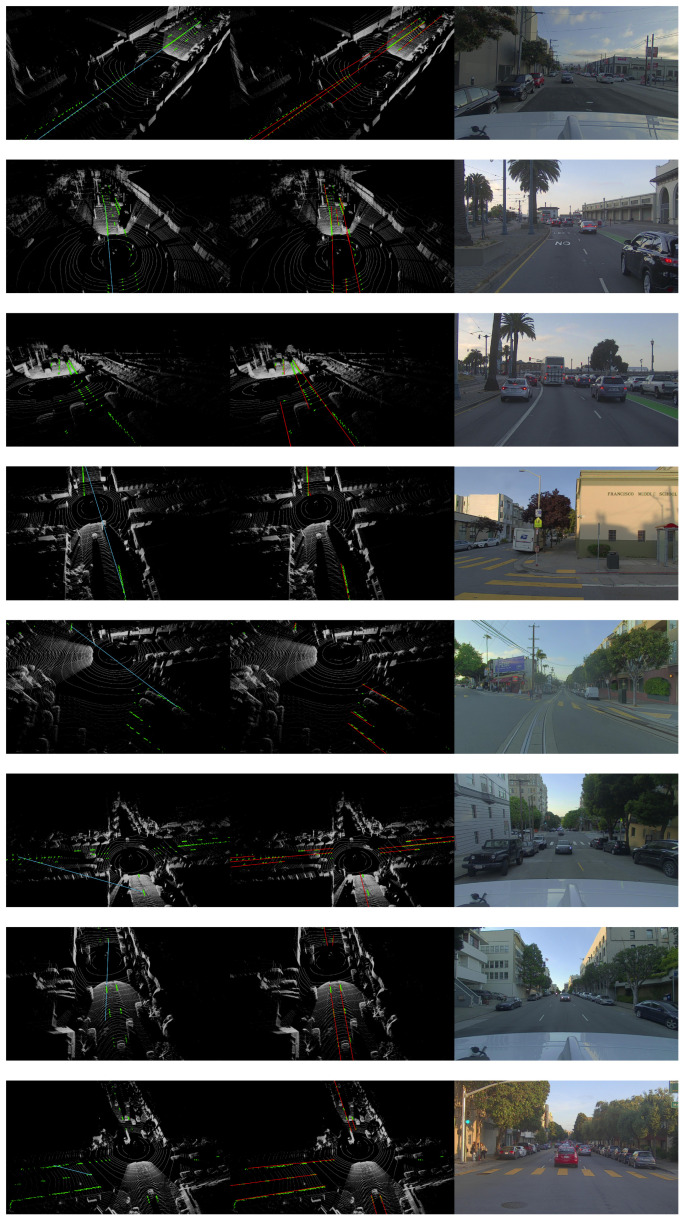
Visualization results for all scenes, from left to right: RANSAC, Ours, RGB.

**Figure 6:**
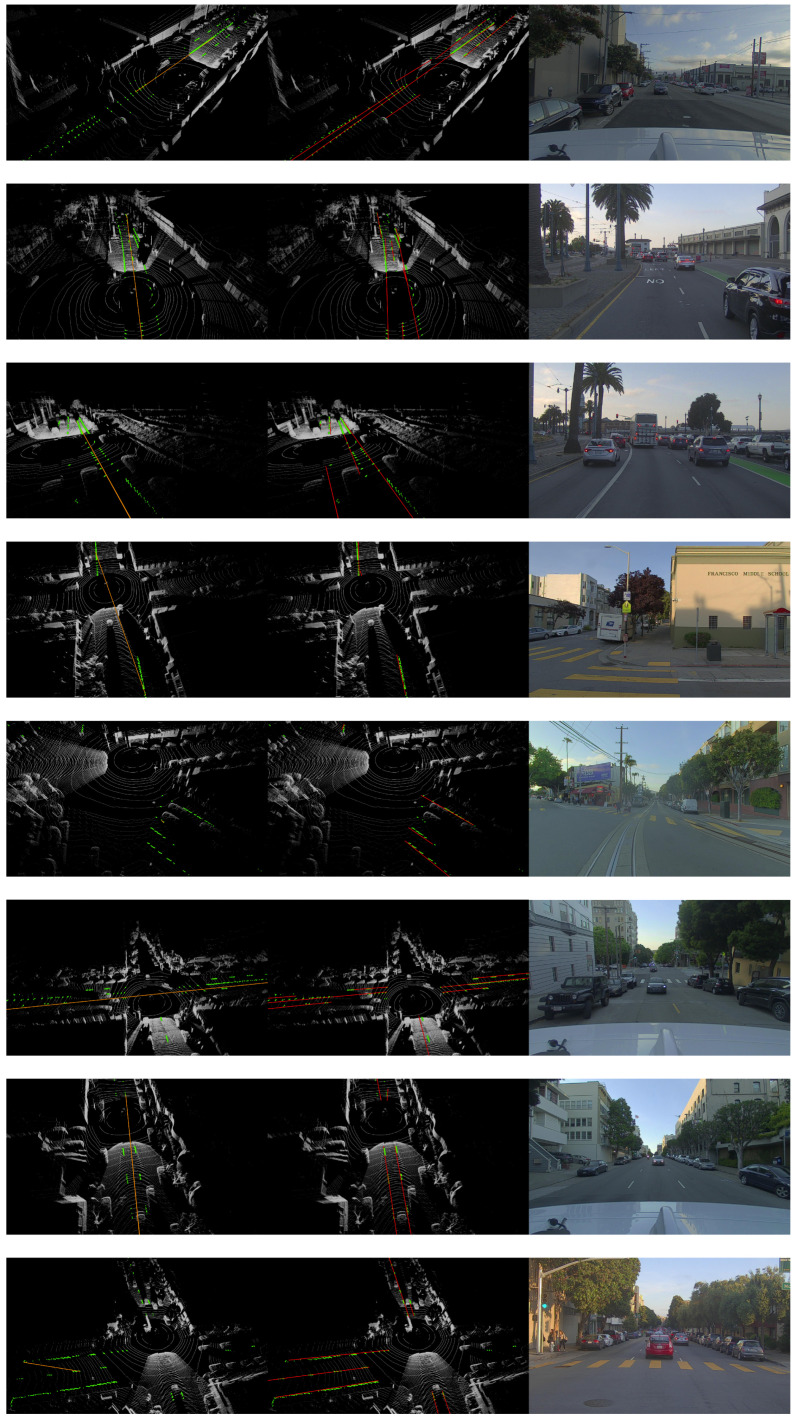
Visualization results for all scenes, from left to right: DSAC, Ours, RGB.

**Figure 7:**
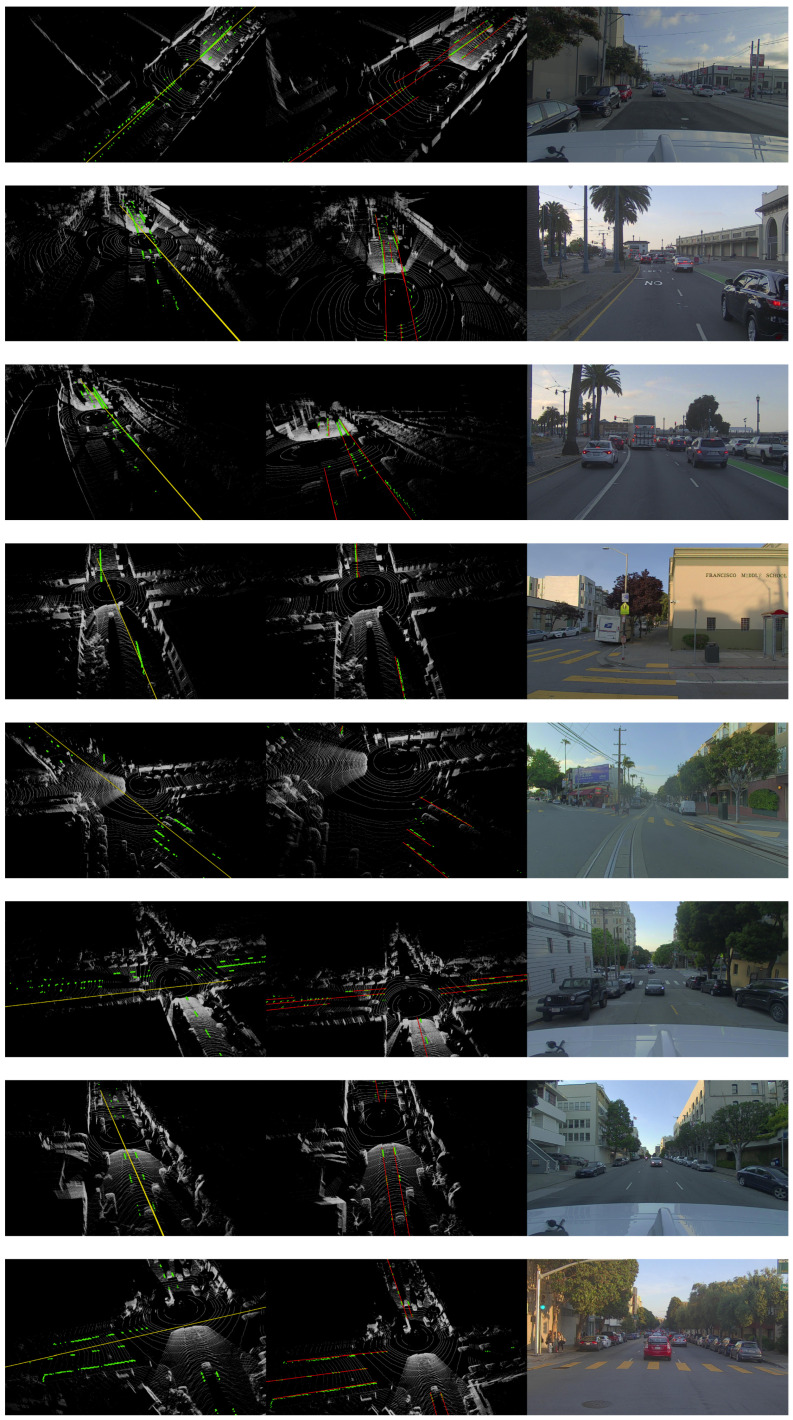
Visualization results for all scenes, from left to right: Poly, Ours, RGB.

We compared the proposed ScatterHough with RANSAC [13], DSAC [30], multiRANSAC [25], and polynomial curve fitting(Poly). Different types of scenes are selected in the experiments, including straight dual-lane, crossroad, fork road, slope road, double dashed line, and curve line. We use 3D point clouds to visualize the fitting results, in which the outputs of different algorithms are denoted by colored lines. An RGB image shows a view of the corresponding scene. Computational results are shown in Figure 4, Figure 5, Figure 6 and Figure 7.

In order to make a numerical comparison, we calculated the number of fitting points and the mean square error for each scenes. The results are reported in Table 1 and Table 2. Here, *inline* indicates the number of the points with the distance to the straight line is less than a pre-set value (0.25 m in this work), *total* is the number of total points, and it should be the same for the specific scene. *accuracy* is calculated as *inline* divided by *total*.

It can be seen that our proposed method can provide a better fit for the lane line in a real-world scenario and obtain the highest *inline* and *accuracy* compared with the other four algorithms. The straight lines in our outputs are parallel to each other, and they do not cross each other, while the outputs of multiRANSAC have obvious differences from the real world lane line in the sense that they occasionally cross over each other. Furthermore, our method shows the strong anti-interference capability, especially when the lane line is partially blocked and the LiDAR point cloud is not comprehensive.

For two lane lines with a significant distance between them, our algorithm can correctly separate them into two independent lines. Our method also takes both the distribution and the density of points into considerations since the unbalanced nature of a LiDAR point cloud can be potentially misleading.

### 4.3. Computational Efficiency

We compared the proposed ScatterHough with the same four algorithms for efficiency. The algorithms were coded in Python, and all the tests were run on the same Intel i9 8950hk 2.6 GHz CPU with 32 GB RAM PC and the Windows 10 operating system. We use frame rate to measure the number of consecutive frames that can be processed for each algorithm. The results are reported in Table 3.

It can be found that a polynomial curve fitting method can achieve the highest FPS because of its nature. However, since it puts a strong constraint on the function prototype, its fitting effect is very poor in complex practical scenarios (see Table 1 and Table 2). Our method achieves the FPS of 12, which outperforms Ransac, Dsac, and multiRansac. In practice, the effective detection range of most LiDARs is within 220 m [21], and since the maximum driving speed of vehicles cannot exceed 250 km/h (approximately 70 m/s) in most countries, such frame rate can fully satisfy the real-time processing need for ADAS.

### 4.4. Hyper-Parameters Setting

**Figure 8:**
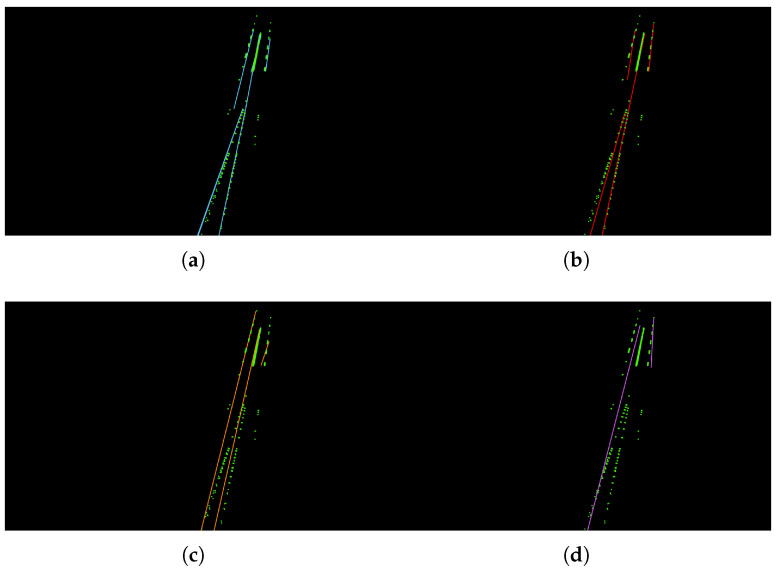
Visualization results for different values of *d*. (**a**) *d* = 0.1; (**b**) *d* = 0.25; (**c**) *d* = 1; (**d**) *d* = 2.

**Figure 9:**
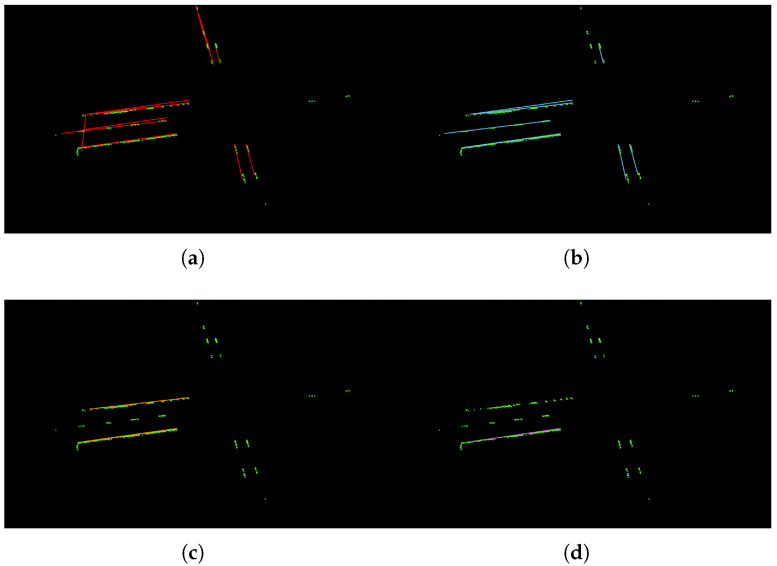
Visualization results for different values of threshold. (**a**) threshold = 10; (**b**) threshold = 30; (**c**) threshold = 60; (**d**) threshold = 120.

**Figure 10:**
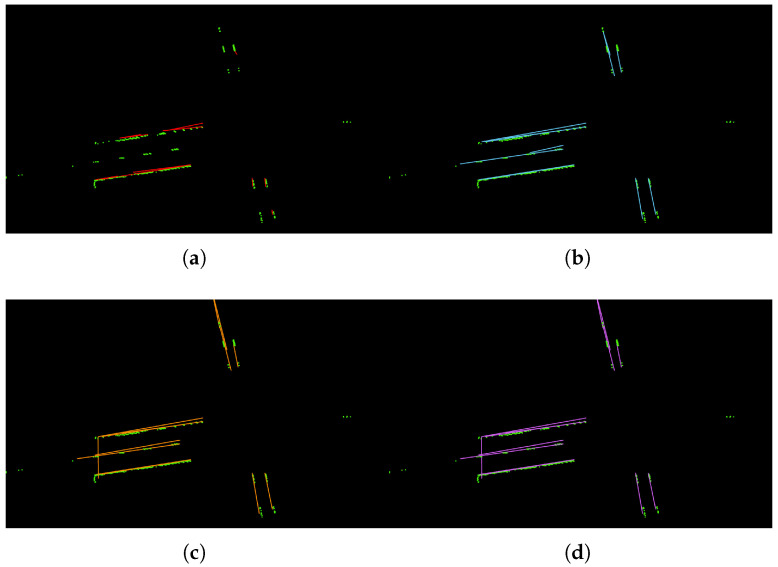
Visualization results for different values of MaxGap. (**a**) MaxGap = 5; (**b**) MaxGap = 10; (**c**) MaxGap = 15; (**d**) MaxGap = 25.

There are three main hyper-parameters in the proposed method: the lane width *d*, the threshold number of the points for fitting a straight line, and the MaxGap between two sections. Different values of each hyper-parameter are analyzed and the visualization results are shown in Figure 8, Figure 9 and Figure 10.

Table 4 shows the comparison results for different values of *d*. We can see that, when *d* = 0.1 or *d* = 0.25, the model can have the better fits for the lane line with higher accuracies. However, when the value of *d* increases, the fitting line obviously deviates from the reality. In most countries, the lane width is within 0.25 m, and we choose d=0.25 as the default value in this work. Table 5 shows the comparison results for different values of threshold. We can see that, when threshold is too small, it can cause the over-fitting problem. For example, in Figure 9a, there is a vertical straight line on the far left, which is obviously not a lane line. Because of the small threshold, a new lane line is mistakenly fitted. In contrast, when threshold is too big, it can cause incomplete results. As shown in Figure 9c,d, the dashed line area is lost. In this work, we use threshold=30 as the default value. The comparison results for different values of MaxGap are reported in Table 6. As we can see, when MaxGap is too small, some certain lane lines cannot be separated (e.g., Figure 10a). When the value of MaxGap increases, longer lines will be fitted. However, when MaxGap is too big, the two sides of the lane line will be connected (e.g., Figure 10c,d). In this work, we set MaxGap=10 as our default value.

### 4.5. Discussion

Our method uses Hough as the prototype. As a result, when fitting multiple line segments at the same time, the lines will not cross. It has the advantages over the randomness algorithms such as RANSAC since the lines may cross if the randomly sampled points come from different lines in the iterative process. In real-world situations, the lane lines can be influenced by vehicles, road works and whether conditions result in partial occlusion. Our proposed method is robust to the incomplete lane line and environmental noises. In addition, our method takes both the number of points and the distribution of points into consideration in order to avoid the misleading problem caused by a high density of points for the same targets. Furthermore, our proposed method is computationally efficient and only requires the small storage space, which is attractive in real automatic driving applications compared to the neural network based models.

## 5. Conclusions

We have proposed an algorithm based on Hough transform to overcome the difficulties in current automatic driving, such as insufficient computing resources and high environmental noise, and have tested the algorithm on the PandaSet benchmark. Compared with previous mainstream algorithms, our algorithm has the advantages of low computational complexity and robustness to environmental noise. The proposed method has the potential to outperform the mainstream neural network methods given that the sensor technology continues to improve in the future.

## Figures and Tables

**Table 1:** Comparison results for all scenes. The best results are indicated in bold.

Scene	Method	Metric	Result
Straight dual-lane line	ScatterHough (Ours)	inline	**665**
		total	821
		accuracy	**0.8090**
	Ransac	inline	533
		total	821
		accuracy	0.6492
	Dsac	inline	280
		total	821
		accuracy	0.3410
	multiRansac	inline	614
		total	821
		accuracy	0.7479
	Poly	inline	216
		total	821
		accuracy	0.2631
3-lane crossroad	ScatterHough (Ours)	inline	**335**
		total	479
		accuracy	**0.6994**
	Ransac	inline	179
		total	479
		accuracy	0.3737
	Dsac	inline	12
		total	479
		accuracy	0.0251
	multiRansac	inline	31
		total	479
		accuracy	0.0647
	Poly	inline	5
		total	479
		accuracy	0.0104
fork road	ScatterHough (Ours)	inline	**90**
		total	178
		accuracy	**0.5056**
	Ransac	inline	50
		total	178
		accuracy	0.2809
	Dsac	inline	3
		total	178
		accuracy	0.0169
	multiRansac	inline	41
		total	178
		accuracy	0.2303
	Poly	inline	0
		total	178
		accuracy	0
slope road	ScatterHough (Ours)	inline	**594**
		total	734
		accuracy	**0.8093**
	Ransac	inline	55
		total	734
		accuracy	0.0749
	Dsac	inline	125
		total	734
		accuracy	0.1703
	multiRansac	inline	162
		total	734
		accuracy	0.2207
	Poly	inline	52
		total	734
		accuracy	0.0708

**Table 2:** Comparison results for all scenes. The best results are indicated in bold.

Scene	Method	Metric	Result
double dashed line	ScatterHough (Ours)	inline	**393**
		total	429
		accuracy	**0.9161**
	Ransac	inline	106
		total	429
		accuracy	0.2471
	Dsac	inline	13
		total	429
		accuracy	0.0303
	multiRansac	inline	305
		total	429
		accuracy	0.7110
	Poly	inline	0
		total	429
		accuracy	0
curve line	ScatterHough (Ours)	inline	**794**
		total	1335
		accuracy	**0.5947**
	Ransac	inline	688
		total	1335
		accuracy	0.5154
	Dsac	inline	44
		total	1335
		accuracy	0.0330
	multiRansac	inline	425
		total	1335
		accuracy	0.3184
	Poly	inline	23
		total	1335
		accuracy	0.0172
Overall	ScatterHough (Ours)	inline	**3926**
		total	5702
		accuracy	**0.6885**
	Ransac	inline	1983
		total	5702
		accuracy	0.3477
	Dsac	inline	854
		total	5702
		accuracy	0.1497
	multiRansac	inline	2124
		total	5702
		accuracy	0.3725
	Poly	inline	356
		total	5702
		accuracy	0.0624

**Table 3:** Efficiency comparison results for different algorithms.

Method	ScatterHough (Ours)	Ransac	Dsac	multiRansac	Poly
frames per second (FPS)	12	8	2	5	313

**Table 4:** Comparison results for different values of *d*. The best results are indicated in bold.

	*d* = 0.1	*d* = 0.25	*d* = 1	*d* = 2
*inline*	769	**802**	129	32
*total*	821	821	821	821
*accuracy*	0.9367	**0.9769**	0.1571	0.0390

**Table 5:** Comparison results for different values of threshold. The best results are indicated in bold.

	Threshold = 10	Threshold = 30	Threshold = 60	Threshold = 120
*inline*	403	**409**	73	12
*total*	429	429	429	429
*accuracy*	0.9394	**0.9534**	0.1702	0.0280

**Table 6:** Comparison results for different values of MaxGap. The best results are indicated in bold.

	MaxGap = 5	MaxGap = 10	MaxGap = 15	MaxGap = 25
*inline*	344	409	**412**	**412**
*total*	429	429	429	429
*accuracy*	0.8019	0.9534	**0.9604**	**0.9604**

## Data Availability

The PandaSet dataset is publicly available from https://scale.com/resources/download/pandaset accessed on 24 March 2022.
